# Dwelling on Differences in Health

**DOI:** 10.1289/ehp.113-a592

**Published:** 2005-09

**Authors:** Jennifer Medlin

While millions of Americans suffer from illnesses such as cardiovascular disease, diabetes, high blood pressure, and cancers of the breast and prostate, these maladies affect certain population groups more than others, for a host of complex reasons suspected but not confirmed by hard scientific data. Observation of these health disparities (defined as differences in incidence, prevalence, mortality, and burden of disease among specific population groups) led the NIH to fund eight different research centers with a total of $60.5 million over five years to study what factors might mediate the onset or outcomes of these common diseases.

Two years ago investigators at the eight Centers for Population Health and Health Disparities (CPHHDs) began the complicated task of sorting out these disparity-inducing influences. Today they are just beginning to evaluate how biological, social, cultural, environmental, and economic factors overlap and combine in such a way as to influence the rate of disease—and in some cases the outcomes of disease—in certain populations.

## Breast Cancer in Chicago’s South Side

Researchers at the University of Chicago CPHHD are focusing primarily on how both mind and body may interact with genes to create breast cancer differences between whites and blacks. Center director Sarah Gehlert and her co-investigators are following women in 15 predominately black neighborhoods on Chicago’s South Side. Although white women have higher incidence of breast cancer, black women have higher mortality from the disease. Gehlert’s colleague, medical professor Funmi Olopade, is helping the group address the question of why black women in both the United States and West Africa develop breast cancers that are more aggressive and more often lethal than tumors in white women, and do so at a younger age.

They hope to answer this question by analyzing the RNA and DNA of breast tumors from women from Ibadan, Nigeria, and the Chicago South Side. According to Olopade, genetic factors contribute to 5–10% of breast cancers overall, but in women younger than 40, they are responsible for about 25% of all breast tumors. In West Africa, breast cancer is considered a disease of young women, with 43 being the average age of diagnosis in Ibadan, some 10–15 years younger than in industrialized countries.

Olopade is examining the molecular characterization of the tumors, while Gehlert is following the women in terms of their psychosocial functioning. Meanwhile, center co-director Martha McClintock is using rats to test the hypothesis that social isolation—and the resulting hypervigilance that isolated animals exhibit—may lead to the development of spontaneous mammary tumors. The investigators suspect that social isolation among women living in dilapidated areas with high rates of violent crime may contribute to poor cancer outcomes.

Gehlert and her colleagues have spent a significant amount of time working to improve access to—and quality of—useful health information for women in the South Side community. To recruit the 503 women who participated in the initial focus groups, “we literally stood at bus stops and church parking lots,” Gehlert recalls. “We asked them about health messages that reach them in the community and found that these messages had been reduced primarily to ‘what not to do,’” instead of useful direction on how to be well for the long term.

Local health advocate and long-time South Side resident Annie Pope agrees: “We found there is an absence of information about breast cancer; these women know more about AIDS than breast cancer,” she says. “They need access to mammograms, but they also need to know how to develop a relationship with their physician.”

## Prostate Cancer Disparities

At the University of Pennsylvania CPHHD, investigators want to find out why black men differ noticeably from white men in incidence of and death from prostate cancer. According to the National Cancer Institute, 73 of every 100,000 black men died from prostate cancer for the period of 1996 to 2000, compared to 30.2 of every 100,000 white men.

Center director Timothy Rebbeck and his colleagues are studying behavioral and environmental factors associated with prostate cancer among black men, including whether genes involved in testosterone metabolism may predict a good or bad prostate cancer outcome. They will also explore possible discrimination in screening practices (reflecting biases of both physicians and patients) as well as treatment after diagnosis, which may affect prostate cancer outcomes. Still another study focuses on social, cultural, behavioral, and environmental factors on quality of life after black and white patients receive their prostate cancer diagnosis.

“Most men develop prostate tumors sooner or later,” Rebbeck points out. “The key is not to figure out who has prostate cancer, but who has a bad outcome and then focus on how to avoid [that outcome].” Center investigators also hope to document how socioeconomic factors (such as neighborhood, income level, and education level) may influence how and whether cancer is treated. “We can guess that [an impoverished environment, lower income, and lower educational attainment] have a negative influence, but it’s not well known,” Rebbeck explains. “We need the data to show if they’re really influencing disease outcome.”

## Cervical Cancer in Ohio Appalachia

Studies under way at The Ohio State University CPHHD target health disparities associated with yet another form of cancer. Center director Electra Paskett and her team of colleagues want to know why cervical cancer incidence is higher among women living in the state’s Appalachian country, which comprises 29 counties. Indeed, cervical cancer rates in this area range from 11.4 to 20.3 cases per 100,000 women, compared with a national rate of 9.6 cases per 100,000 women.

The goal of one study is to boost early detection of cervical cancer (which increases rate of survival) by increasing the proportion of women in this area aged 18 and older who receive regular Pap smears and return for follow-up care if necessary. Because smoking, a known cervical cancer risk, is more common among this population than in the general U.S. population, a second study will test the effectiveness of a smoking cessation program. A third study of 1,200 women will explore how variables such as behavior, economic conditions, and barriers to health care, as well as biological factors (including infection with the sexually transmitted human papillomavirus, which may be a precursor to cervical cancer) may interact to contribute to cervical abnormalities.

Paskett says the greatest challenge so far has been recruiting participants across such a large geographic area as well as outfitting the 16 participating clinics with resources to support the study. “We’ve had to provide clinics with special equipment they didn’t have, such as centrifuges and freezers,” she explains. “Some of the clinics were short-staffed and not up to date with computer technology.”

## When Many Diseases Burden One Population

Research under way at the Wayne State University Center for Urban and African American Health targets several health problems burdening the black population in the Detroit area. Three research projects share the common themes of obesity, diet, lifestyle factors such as physical activity, and obesity-related cancer and cardiovascular disease. According to center director John Flack, blacks as a group have higher incidence not only of cancer, but also of high blood pressure, stroke, kidney disease, and obesity; they also tend to exercise less.

A team lead by Zora Djuric is seeking to better understand why weight gain is more common in black breast cancer survivors than in whites, a statistic of great concern because weight gain is linked to breast cancer recurrence. Another study explores the link between sodium intake (and a possible resulting rise in blood pressure) and weight gain. The researchers also hope to find optimal ways to improve outcomes of black patients undergoing cardiovascular rehabilitation.

Although Flack suspects that environmental differences and lower birth weights in black babies may contribute to the disparity in health problems among this population compared to whites, he cautions against assigning too much importance to the role of genetics. “Most genetic variations do not occur between ethnic groups,” he says. “My guess is that the genetic contribution is much smaller than some have speculated.”

## Boston Puerto Ricans Beleaguered by Stress

Researchers at the Tufts University CPHHD aim to uncover the factors that make older Puerto Ricans living in the greater Boston area significantly more likely to suffer from physical disability, depression, cognitive impairment, type 2 diabetes mellitus, and other chronic health conditions than do non-Hispanic white elders living in the same neighborhoods. Center director Katherine Tucker suspects that higher levels of stress—possibly resulting from poverty, migration, acculturation (including adopting a U.S. diet), and perceived discrimination—leads to greater long-term physical expressions of stress (or “allostatic load”) and eventually adverse health outcomes. Investigators are collaborating with a community organization, La Alianza Hispana, to offer social intervention, health care, and nutrition information to local communities.

So far, investigators have completed more than 400 interviews with Puerto Rican adults aged 50–75 living in the Boston area and have gathered information on poverty, language isolation, urban environments, nutritional intake, and measures of stress. The latter was assessed both by questionnaires and by physiological measures, such as levels of stress hormones including the catecholamines and cortisol. The investigators plan to follow up with two forms of intervention—one group will receive multivitamins and compliance reminders, while the other will receive social interaction activities to relieve stress. Each intervention will continue for two years, and postintervention measures of health will be compared to baseline for these groups in relation to the remaining participants who did not receive the interventions.

A companion study is under way to assess genetic interactions with diet on the risk of cardiovascular disease and diabetes. According to Tucker, 40% of Puerto Rican immigrants aged 60 or older have type 2 diabetes, compared with fewer than 20% of whites, numbers that Tucker says are “out of control.” Though their long-term goal is to document disparities and provide information to target improved services that will promote health in this high-risk group, the researchers have seen some immediate benefits already. “We’re having a very positive response from patients who didn’t know they were ill and who have followed up with their physicians to start a treatment plan,” Tucker says.

## Health Disparity: A Positive Thing?

In at least one case, belonging to a minority group may confer health benefits. That’s the thinking behind what James Goodwin, director of the CPHHD at the University of Texas Medical Branch in Galveston, calls “the Hispanic paradox,” a finding that the health of many Hispanic populations in the United States is similar to that of whites, even though the Hispanic groups suffer disadvantages in income, health insurance, housing, education, and other factors that correlate strongly with health.

For Hispanics, health varies in relation to neighborhood composition. Hispanics living in largely homogeneous census tracts enjoy lower cancer incidence and lower cancer mortality than those living in neighborhoods with low percentages of Hispanics. For example, data from the Hispanic Established Populations for Epidemiologic Studies of the Elderly, a population-based longitudinal study of 3,050 older Mexican Americans living in the Southwest, showed a more than threefold difference in cancer prevalence among the subjects as a function of the percentage of Mexican Americans in their respective census tracts. Goodwin and his colleagues want to first find out what it is about high-density Hispanic neighborhoods that promotes good health, as well as the pathways or mechanisms that seem to transmit good health to residents of those neighborhoods. They suspect nutrition and buffers against stress both play some role.

“The role of stress in disease has been way, way underestimated and ignored,” Goodwin says. “As doctors, we tend to focus only on things we can measure. We don’t have a stress-o-meter, so we can’t easily measure stress. We focus on what we can objectify—pollution, for example, as opposed to *concerns* about pollution.”

The researchers are merging readily available data sets—including the Hispanic Established Populations for Epidemiologic Studies of the Elderly as well as the Surveillance, Epidemiology, and End Results cancer registry—with census data, and then analyzing them to better understand the role of neighborhood in cancer incidence.

## Neighborhoods and Negative Health Influences

Researchers at the RAND CPHHD in Santa Monica have also noted how neighborhoods can affect health outcomes, including infant mortality, life expectancy, and the development of chronic diseases such as heart disease and asthma. Center director Nicole Lurie says it may be possible to understand how neighborhood environments influence the development of disease by examining predisease markers of cumulative biological stress, including hormonal reactions and inflammatory and endocrine markers.

A major accomplishment for this center has been the development of the Contextual Data Library, a core data library for use in future studies. Researchers have layered publicly available health, socioeconomic, and census data with segregation and cost-of-living indices as well as measures of street connectivity, air pollution, and land use. The result is a detailed look, statistically speaking, of any given individual’s neighborhood characteristics. The data can be downloaded for free (see http://www.rand.org/labor/aging/dataprod/cdl/listdata.html).

Studies planned or under way at the RAND center will evaluate numerous neighborhood variables, including how the presence of parks shapes physical activity and health; whether different types of neighborhoods and neighborhood features produce different biological “footprints” (patterns of biological markers); how elements of the built environment may influence mental health; how physical and social aspects of a neighborhood may contribute to the disabling process in the elderly; whether neighborhood characteristics correlate with obesity, physical activity, and diet; and how outdoor air pollution affects the worsening of asthma.

“There continues to be a major debate about what factors make people sick,” Lurie says. “We want to find out how much is caused by neighborhood factors, particularly those that could be modified by public policy.”

## Neighborhood Effects on Breast Cancer

The impact of neighborhoods on breast cancer is the focus of research under way at the CPHHD at The University of Illinois at Chicago. C enter director Richard Warnecke and his colleagues are studying the relationship between patients’ social environment and access to early detection and diagnosis of breast cancer in black, Hispanic, and white women. According to Warnecke, stage at diagnosis is the best predictor of survival.

Related studies will examine the influence of social networks on patients’ use of health care services and response to symptoms, identify factors and beliefs that may delay a patient’s seeking medical attention (for example, the fear that touching the breast too often or getting too many mammograms will itself cause cancer), and explore factors from discovery through treatment that influence breast cancer prognosis.

Investigators are evaluating data from the state breast cancer registry coded by census variables and information about where individual participants live, collecting blood samples to analyze DNA markers and stress measures, and conducting extensive interviews with both patients themselves and members of each patient’s primary social support network.

Long term, Warnecke hopes the studies will have a positive “systems effect” on breast cancer screening and treatment. “In Chicago, if you’re poor, there can be as much as a six-month waiting time for screening; if an anomaly is found, there can be a [further] six-month waiting time for a biopsy,” he says. The reason for this lag time is the lack of facilities that are accessible at a cost that poor women can afford. The wait could, for some women, mean the difference between life and death.

Like Warnecke, scientists working at all the CPHHDs hope not only for answers to their questions about disease causes and interventions at both the population and individual levels, but also for the tools to promote change and significantly reduce health disparities altogether.

## Figures and Tables

**Figure:**
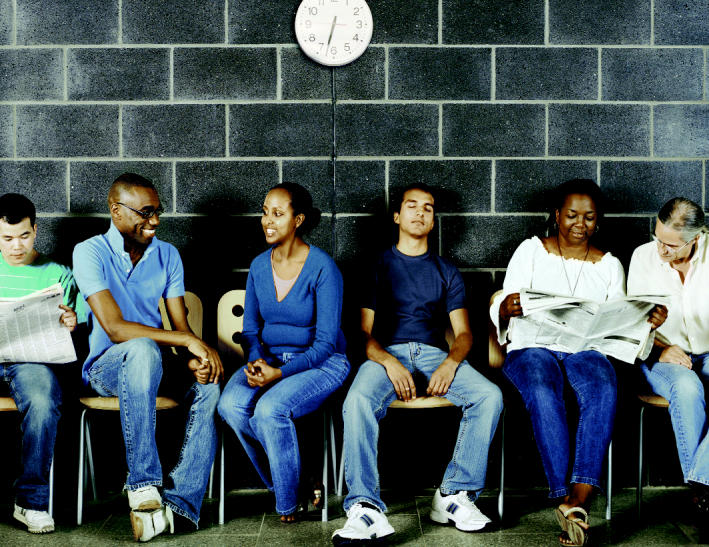
Pieces of the puzzle. The NIEHS Centers for Population Health and Health Disparities are working to uncover why the factors that make populations unique may also work to make them more—or less—vulnerable to environmentally related disease.

**Figure:**
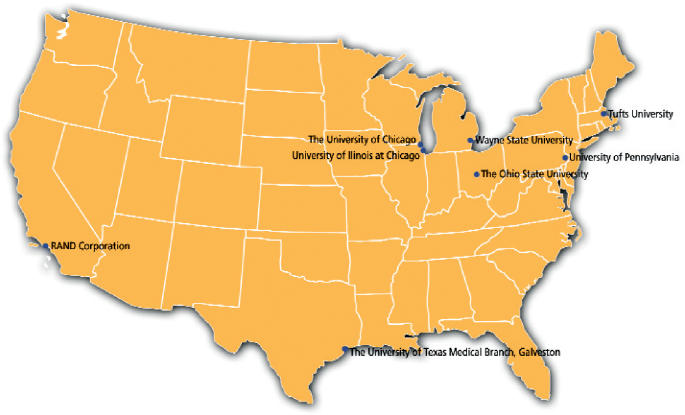
Connecting the dots. Researchers at the eight centers are studying how biological, sociocultural, environmental, and economic factors combine to contribute to disparities in disease among local populations across the United States.

